# Cowpox Virus in Llama, Italy

**DOI:** 10.3201/eid1708.101912

**Published:** 2011-08

**Authors:** Giusy Cardeti, Alberto Brozzi, Claudia Eleni, Nicola Polici, Gianlorenzo D’Alterio, Fabrizio Carletti, Maria Teresa Scicluna, Concetta Castilletti, Maria R. Capobianchi, Antonino Di Caro, Gian Luca Autorino, Demetrio Amaddeo

**Affiliations:** Author affiliations: Istituto Zooprofilattico Sperimentale delle Regioni Lazio e Toscana, Rome, Italy (G. Cardeti, A. Brozzi, C. Eleni, M.T. Scicluna, G.L. Autorino, D. Amaddeo);; Azienda Unità Sanitaria Locale, Viterbo, Italy (N. Polici);; Ambulatorio Veterinario Farnese, Farnese, Italy (G. D’Alterio);; National Institute for Infectious Diseases, Rome (F. Carletti, C. Castilletti, M.R. Capobianchi, A. Di Caro)

**Keywords:** viruses, agriculture, llama, lama glama, Italy, cowpox, CPVX, serology, dispatch

## Abstract

Cowpox virus (CPXV) was isolated from skin lesions of a llama on a farm in Italy. Transmission electron microscopy showed brick-shaped particles consistent with orthopoxviruses. CPXV-antibodies were detected in llama and human serum samples; a CPXV isolate had a hemagglutinin sequence identical to CPXV-MonKre08/1–2-3 strains isolated from banded mongooses in Germany.

The llama (*Lama glama*) is a South American camelid used as a pack and meat animal by Andean cultures since pre-Hispanic times. Today, llama breeding is spreading in North America where the animals are used for wool production and as livestock guards. In Italy, llamas are raised in the northern and central regions to produce meat and wool, but they are more commonly considered companion animals or used as pack animals for trekking tours in the mountains.

Viral diseases of llamas are becoming better known as a result of extensive research in North America (*1*) prompted by the recent growth in commercial breeding of New World camelids. Many of the viral diseases that affect camelids are related to bovine, equine, ovine, and swine virus infections. When examining skin lesions on llamas, viral diseases to consider as differential diagnoses include vesicular stomatitis, rabies, poxvirus (contagious ecthyma and cowpox virus [CPXV]) (*2*), foot-and-mouth disease, bluetongue, and mucocutaneous fibropapillomas (*3*).

In July 2009, five of 7 llamas at a farm near Calcata (Viterbo) in Northern Latium, Italy, had skin lesions at different sites (palpebral conjunctiva, auricles, teats, mouth, and anus) that evolved from nodules to crusts; some had a crater morphologic appearance typical of poxvirus lesions (Figure 1, panel A). Within 10 days, 2 males showed depression and lethargy, anorexia, and recumbency until death. A short time later, another llama, a 7-year-old female, became ill and was euthanized. Necropsy was conducted on the euthanized animal, and samples were collected for laboratory investigation. No other animals belonging to numerous species of birds (local and exotic) and mammals (goats, cattle, swine, donkeys, and horses) living at the farm showed any of the above-mentioned symptoms.

**Figure 1:**
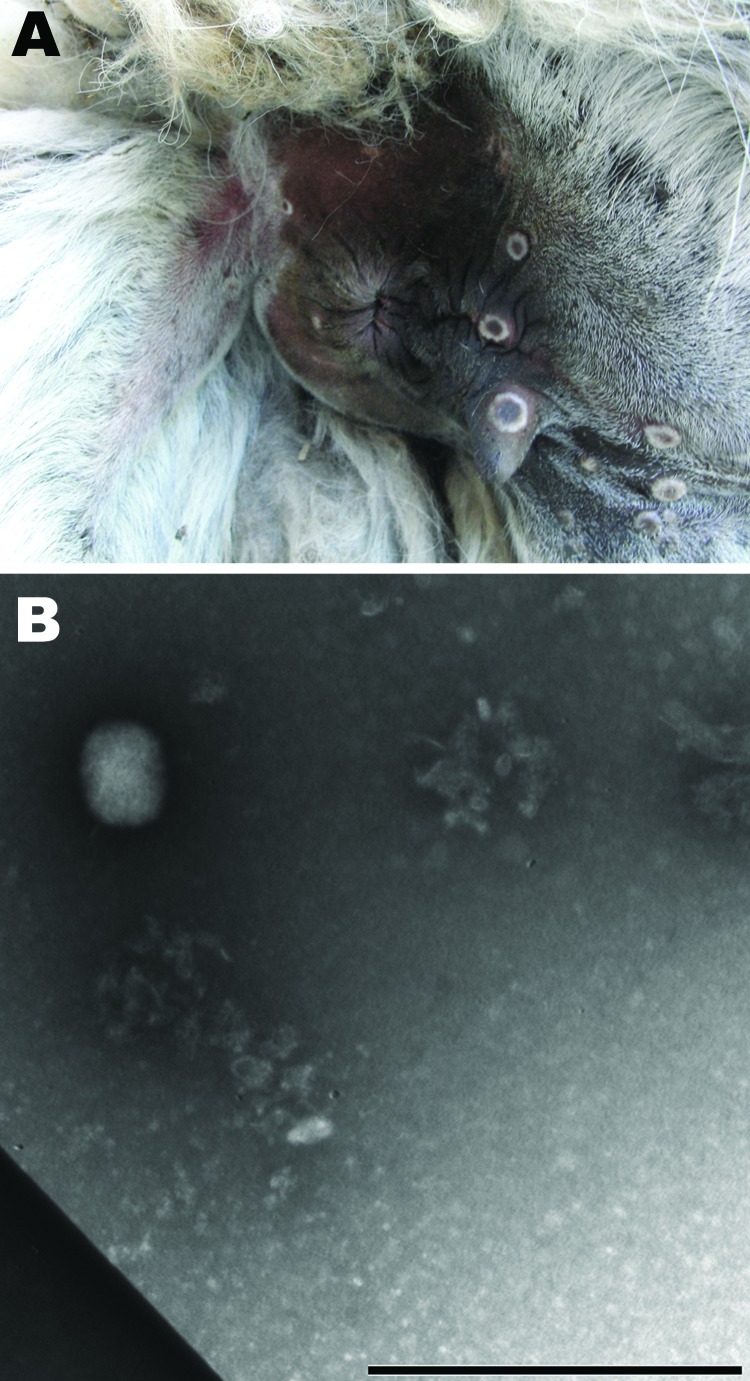
A) Skin lesions showing a crater morphologic appearance typical of poxvirus around the anus of a llama from a farm near Calcata (Viterbo) in Northern Latium, Italy. B) Electron micrography image of skin lesion sample showing negatively stained brick viral particle of ≈160–220 nm, consistent with orthopoxvirus. Scale bar = 1 µm.

Because mice and rats are considered carriers of CPXV (*4*) and birds of prey at the farm were fed frozen mice imported from a large farm in Germany, 2 lots of these mice were sampled (31 animals in total) along with 44 wild gray rats (*Rattus rattus*) captured on the farm for molecular analysis and virus isolation. Blood samples were collected for serologic analysis from 4 cattle, 25 goats, 18 donkeys, 3 horses, the 4 living llamas, and 4 humans (the farmer and 3 workers).

At necropsy, mandibular lymphoadenopathy; hemorrhagic gastroenteritis; and congestion of stomach, spleen, and kidney were observed in the euthanized female llama, signs consistent with a bacterial infection. In fact, a strain of *Salmonella enterica* serovar Typhimurium was isolated from lung, liver, spleen, kidney, and lymph node samples from the dead female llama and from chicken feces.

Samples of skin lesions were fixed in 10% buffered formalin, embedded in paraffin, cut 4 µm thick, and stained with hematoxylin and eosin. Histologically, the skin lesions showed eosinophilic granular intracytoplasmic inclusion bodies in basal and spinous layers of epidermis, compatible with poxvirus infection; superficial ulceration and extensive intraepidermal abscesses were also found.

Because we suspected a poxvirus, the skin lesions were processed (*5*) for electron microscopy techniques. Brick particles of ≈160–220 nm were observed, consistent with orthopoxvirus (Figure 1, panel B).

Two mammal cell lines (Vero and BHK_21_) were used for virus isolation from skin lesions of the euthanized animal. During incubation of the cell cultures at 37°C, focal areas of cytopathogenic effect appeared within 3 days from injection, with complete lysis of the monostrate within 5 days. All the samples from mice and rats (pool of liver, spleen, and kidney) had negative results after 3 passages on both cell lines.

Total viral DNA was extracted from homogenized crusts and Vero cells supernatant by using conventional chemistry (QIAamp DNA Mini Kit; QIAGEN, Hilden, Germany) according to the manufacturer’s instructions. Five μL of DNA was used as template for a 20 μL total volume real-time PCR (*6*) targeting a region of the cytokine response–modifying protein B (*crmB*) gene, identifying an orthopoxvirus. A wider region of *crmB* (*7*) was amplified by conventional PCR and sequenced to confirm orthopoxvirus identity (data not shown). Phylogenetic analysis was performed by using a complete gene of the hemagglutinin sequence. The phylogenetic tree shows that the llama hemagglutinin sequence is identical to clusters with the cowpox virus strains isolated in Germany in 2008 (CPXV-MonKre08/1–2-3) (Figure 2).

**Figure 2:**
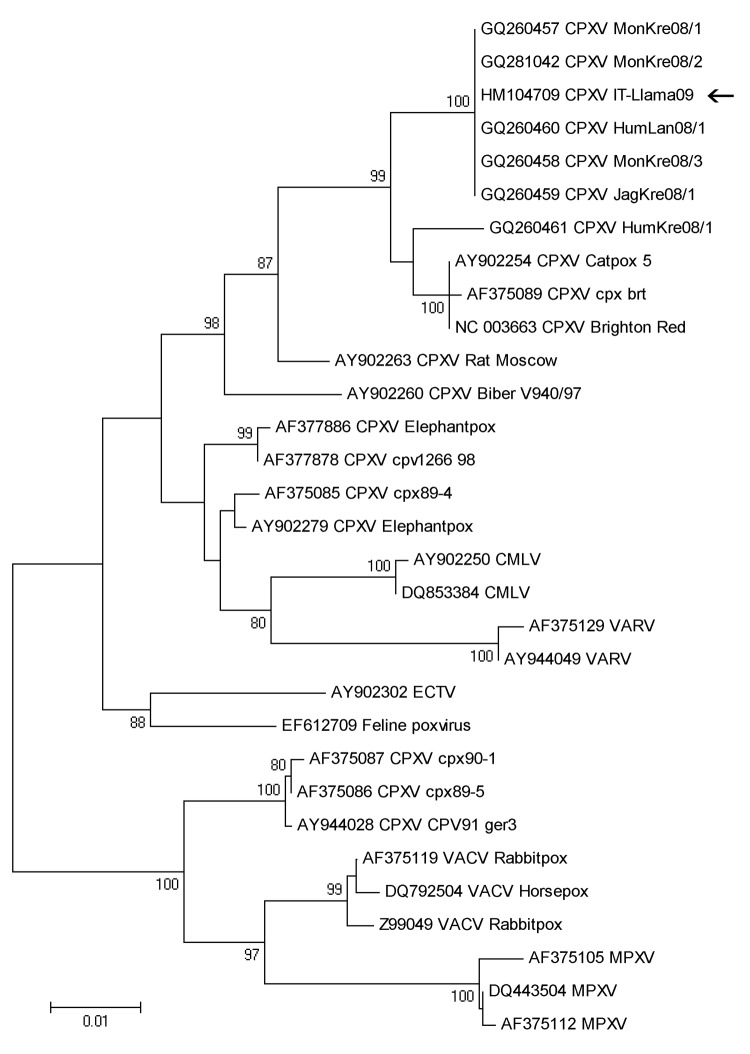
Phylogenetic tree based on nucleotide sequences of the complete hemagglutinin open reading frame (921 bp) from the llama orthopoxvirus isolate (arrow) and additional orthopoxvirus sequences available in GenBank. The tree has been constructed by using nucleotide alignment, the Kimura 2-parameter algorithm, and the neighbor-joining method implemented in MEGA4.1 software (www.megasoftware.net). Bootstrap values >75 are shown at nodes. CPXV, cowpox virus; CMLV, camelpox virus; VARV, variola virus; ECTV, ectromelia virus; VACV, vaccinia virus; MPXV, monkeypox virus. Scale bar indicates genetic diversity at the nucleotide level.

A stock virus suspension of the viral strain isolated from the llama was produced and titrated on Vero cells to use as viral antigen in an in-house seroneutralization (SN) test. The blood of the 4 surviving llamas collected 19 days after the female llama’s euthanasia was assayed by CPXV SN test, as well as for antibodies against bluetongue virus (by ELISA), infectious rhinotracheitis virus (ELISA), *Brucella* (Rose Bengal plate test), and neosporidia (ELISA), all with negative findings. The SN test for detection of CPXV antibodies was also conducted in llama serum collected ≈3.5 months later (day 105), in parallel with samples collected from various animal species (bovine, equine, and caprine). Only the 4 llama samples collected at day 105 showed CPXV antibodies (Table). The antibody titers (immunoglobulin [Ig] G and IgM) in the farm personnel serum samples were determined by indirect immunofluorescence assay by using in-house slides with Vero-E6 cells infected with a Lancy-Vaxina smallpox vaccine virus. Serum from a person vaccinated 4 years previously was used as a positive control. All serum samples had negative results for IgM (<20), while 3 persons had different IgG titers (Table). Only 1 of the 3 persons (titer 640) had been previously vaccinated against smallpox.

**Table:** Results of seroneutralization test for cowpox virus and IFA of serum samples from livestock and humans living on a farm, Italy*

Sample source	No. positive/no. tested		Serum titers, dilution
	Seroneutralization	IFA (IgG)	
Llamas†			
Day 19	0/4	ND	ND
Day 105	4/4	ND	64; 11; 4; 32
Cattle	0/4	ND	
Goats	0/25	ND	
Horses	0/3	ND	
Donkeys	0/18	ND	
Humans	ND	3/4	640; 20; 80

*IFA, immunofluorescence assay; Ig, immunoglobulin; ND, not done. †Days shown are after a female llama at the farm was euthanized.

## Conclusions

Following this outbreak, no other clinical signs have been described among livestock and humans at the farm and no other reports of CPXV have been recorded in Italy. The owner stated all the llamas were born on the farm and had had no contact with exotic mammals. Because the identified strain is apparently identical to the German 2008 mongoose isolates (CPXV-MonKre08/1–2-3) (*8**,**9*), it is tempting to hypothesize that the virus was introduced to the farm through frozen rats used as food for birds of prey. Such rats were sold by a German distributor throughout Europe, which also sold infected rats to the zoo where the identical mongoose CPXV isolate has been described (A. Kurth, pers. comm.).

Cowpox virus is distributed in Europe, western Russia, and adjacent areas of northern and central Asia, with an increasing number of reports in Europe (*10*). In Germany, CPXV has been found in animals in zoos. More recently, CPXV infections have been reported in mongooses (*8**,**9*). CPXV has been recently isolated from 2 cats and persons in contact with them in northern Italy (*6*). To date, no additional reports on the spread of this virus in Italy are available, although it is becoming increasingly popular to own wild and exotic pets and a wide range of recognized wild and domestic animal CPXV-hosts is increasing.

CPXV is responsible for human cowpox, a rare zoonotic infection, which is a self-limiting disease except in immunocompromised and eczematous patients, particularly children, in whom it can become severe. Furthermore, numerous reports of human cowpox affecting young people in Europe indicate that the lack of smallpox vaccination, stopped in 1977, may render people more susceptible to CPXV. For these reasons, CPXV is considered a pathogen of public health importance.
